# Melatonin attenuates scopolamine‐induced cognitive dysfunction through SIRT1/IRE1α/XBP1 pathway

**DOI:** 10.1111/cns.14891

**Published:** 2024-07-26

**Authors:** Xiao‐Qi Liu, Shun Huang, Jia‐Yi Zheng, Can Wan, Tian Hu, Ye‐Feng Cai, Qi Wang, Shi‐Jie Zhang

**Affiliations:** ^1^ State Key Laboratory of Traditional Chinese Medicine Syndrome The Second Affiliated Hospital of Guangzhou University of Chinese Medicine Guangzhou China; ^2^ Department of Neurology The Second Affiliated Hospital of Guangzhou University of Chinese Medicine Guangzhou China; ^3^ Department of Neurology Guangdong Provincial Hospital of Chinese Medicine Guangzhou China; ^4^ Guangdong Provincial Key Laboratory of Research on Emergency in TCM Guangzhou Guangzhou China; ^5^ Department of Nuclear Medicine, The Tenth Affiliated Hospital Southern Medical University (Dongguan People's Hospital) Dongguan China; ^6^ Nanfang PET Center, Nanfang Hospital Southern Medical University Guangzhou China; ^7^ Science and Technology Innovation Center Guangzhou University of Chinese Medicine Guangzhou China

**Keywords:** cognitive impairment, ER stress, melatonin, scopolamine, SIRT1

## Abstract

**Background:**

The prevalence of dementia around the world is increasing, and these patients are more likely to have cognitive impairments, mood and anxiety disorders (depression, anxiety, and panic disorder), and attention deficit disorders over their lifetime. Previous studies have proven that melatonin could improve memory loss, but its specific mechanism is still confused.

**Methods:**

In this study, we used in vivo and in vitro models to examine the neuroprotective effect of melatonin on scopolamine (SCOP)‐induced cognitive dysfunction. The behavioral tests were performed. ^18^F‐FDG PET imaging was used to assess the metabolism of the brain. Protein expressions were determined through kit detection, Western blot, and immunofluorescence. Nissl staining was conducted to reflect neurodegeneration. MTT assay and RNAi transfection were applied to perform the in vitro experiments.

**Results:**

We found that melatonin could ameliorate SCOP‐induced cognitive dysfunction and relieve anxious‐like behaviors or HT22 cell damage. ^18^F‐FDG PET‐CT results showed that melatonin could improve cerebral glucose uptake in SCOP‐treated mice. Melatonin restored the cholinergic function, increased the expressions of neurotrophic factors, and ameliorated oxidative stress in the brain of SCOP‐treated mice. In addition, melatonin upregulated the expression of silent information regulator 1 (SIRT1), which further relieved endoplasmic reticulum (ER) stress by decreasing the expression of phosphorylate inositol‐requiring enzyme (p‐IRE1α) and its downstream, X‐box binding protein 1 (XBP1).

**Conclusions:**

These results indicated that melatonin could ameliorate SCOP‐induced cognitive dysfunction through the SIRT1/IRE1α/XBP1 pathway. SIRT1 might be the critical target of melatonin in the treatment of dementia.

## Graphical abstract

Melatonin is a hormone produced in the pineal gland, for adjusting the circadian rhythm of a vertebrate, particularly a mammal. In this study, it may ameliorate SCOP‐induced cognitive dysfunction through SIRT1/IRE1α/XBP1 pathway. SIRT1 might be the key target of melatonin in the treatment of dementia.

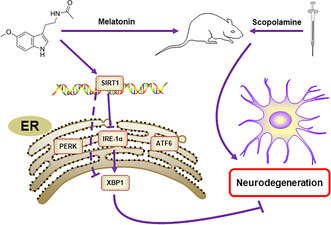

## 1 BACKGROUND

Amnesia is manifested as a deficit in memory, which is one of the early symptoms of aging, mainly caused by brain damage or neurodegenerative diseases, such as mild cognitive impairment (MCI) or Alzheimer's disease (AD).^1^ Amnesia patients are also commonly accompanied by emotional disorders, such as depression, anxiety, and emotional apathy.^2^ It can also be caused temporarily by drugs. Scopolamine (SCOP) has been extensively used to induce cognitive impairment in healthy animals to assess potential therapeutic drugs for some neurodegenerative diseases.^3^ Our previous studies have shown that subcutaneous injection of SCOP can cause damage in the cholinergic system, neural impairment, loss of neurotrophic factors, and oxidative stress damage in mice.^4^ In addition, the effects of SCOP on endoplasmic reticulum (ER)‐related proteins have been reported, and treatment of SCOP on mice activated ER chaperone protein, which could trigger ER stress.^5^, ^6^

Recent studies have shown that the memory loss caused by SCOP might be related to the Sirtuins (SIRTs) family.^7^, ^8^, ^9^ SIRTs family proteins, a class of NAD^+^‐dependent deacetylase in mammalian,^10^ can make various proteins occur in deacetylation and further participate in DNA damage repair, and transcriptional regulation of genes, apoptosis, metabolism, and aging.^11^ Increasing evidence shows that the SIRTs family (the silent information regulator 1–7, SIRT1 to SIRT7) participates in our health and disease,^12^ especially in cognitive dysfunction. For example, SIRT2,^13^ SIRT3,^14^, ^15^ and SIRT7^16^ act as a regulator of neuronal synaptic injury and cognitive deficits. SIRT4 plays a beneficial role in treating pancreatic cancer in vivo and in vitro.^17^ SIRT5 contributes to microglia‐induced neuroinflammation and neuronal damage in ischemic stroke,^18^ while SIRT6 supports microglia protection in postoperative cognitive dysfunction (POCD).^19^

SIRT1 plays a vital role in maintaining cognitive functions, strengthening synaptic plasticity, balancing neuronal metabolism,^20^, ^21^ emotion regulation,^22^ and neuroinflammation improvement.^23^, ^24^ It is also involved in regulating neuronal differentiation,^25^ preventing axonal degeneration,^26^ and inhibiting oxidative stress in neurons.^27^ SIRT1 is indispensable for neuronal protection in mouse models of cognitive disorders, including aging^28^ and AD.^29^ It has been showed that neural apoptosis could lead to ER stress.^30^ Conversely, chronic ER stress not only leads to neurodegeneration but also implicates cognition and memory on account of suppressing the synthesis of synaptic proteins.^31^ The ER stress‐related proteins can be detected in the brain tissue of animal models with cognitive deficits.^32^ Our previous studies have shown that SIRT1 can improve diabetic encephalopathy in db/db mice by inhibiting the ER stress pathway.^33^ However, it is still unclear whether SCOP‐induced ER stress could be inhibited in a SIRT1‐dependent manner.

Melatonin is a hormone produced in the pineal gland to adjust the circadian rhythm of a vertebrate, particularly a mammal.^34^, ^35^ It has been suggested that circadian disorders can contribute to depression and become the basis for melatonin or melatonin receptor agonist efficacy in depression.^36^ The pineal gland of older adults gradually shrunk, and melatonin secretion decreased accordingly. Insufficient melatonin required by various organs in the body leads to aging and cardiovascular diseases.^37^ Accumulating studies have indicated that melatonin could attenuate ER stress. Melatonin might protect BMSC mitochondria against oxidative stress‐mediated injury via the AMPK‐ER stress pathway.^38^ In septic cardiomyopathy, melatonin could upregulate BAP1 to suppress ER stress and mitochondrial damage.^39^ While focusing on neuroprotective effects in cognitive impairment, the specific mechanism of melatonin still needs to be clarified. Interestingly, the family of SIRTs, also closely related to aging, seems to be inextricably linked with melatonin. Among the SIRTs, Melatonin aimed at SIRT3 to alleviate acute kidney injury and small‐intestine injury caused by sepsis,^40^, ^41^ as well as aimed at SIRT2 to improve advanced maternal age‐associated meiotic defects in oocytes.^42^ SIRT6 might be the target point for diabetic cardiomyopathy (DCM).^43^ Referring to recent references, SIRT1 is closely related to melatonin in aging and cancer.^44^, ^45^ Melatonin was reported to control circadian rhythms via upregulating SIRT1 in the senescence‐accelerated prone 8 (SAMP8) mice.^46^ Therefore, we hypothesized that melatonin improved SCOP‐induced cognitive impairment through inhibiting SIRT1‐mediated ER stress.

In this study, we employed SCOP‐induced cognitive dysfunction models in vivo and in vitro to evaluate the effect of melatonin on improving cognitive impairment and relieving anxiety‐like behaviors. In addition, by using EX527 (a specific inhibitor of SIRT1) in in vivo as well as EX527 and *Sirt1* RNAi in in vitro, we found that the SIRT1‐mediated IRE1α/XBP1 pathway was a novel regulatory mechanism of melatonin in protecting against SCOP‐induced cognitive dysfunction.

## 2 MATERIALS AND METHODS

### 2.1 Reagents

Nissl Staining Solution, Antifade Mounting Medium with DAPI, and RIPA Lysis Buffer were purchased from Beyotime Biotechnology (Shanghai, China). Melatonin and EX527 were obtained from MedChemExpress, LLC (Princeton, NJ, USA). Scopolamine Hydrobromide, MTT, and tunicamycin were purchased from Sigma‐Aldrich (Saint Louis, MO, USA). Nanjing Jiancheng Bioengineering Institute (Nanjing, China) provided the following kits: ChAT, Ach, MDA, SOD, GSH‐Px, and CAT. The AChE kit was purchased from Beijing Solarbio Science & Technology Co., Ltd. (Beijing, China). RNAiso Plus, TB Green Premix Ex Taq, and Prime Script RT Master Mix were obtained from Takara Bio Inc. (Otsu, Shiga, Japan). Lipofectamine 3000 transfection reagent and anti‐p‐PERK antibody were purchased from Invitrogen Corporation (Carlsbad, CA, USA). The *Sirt1* RNAi was synthesized by Guangzhou RiboBio Co., Ltd. (Guangzhou, China). Cell Signaling Technology, Inc. (Danvers, MA, USA) offered the following antibodies: anti‐SIRT1, anti‐Bip, anti‐PDI, anti‐PERK, anti‐IRE1α, anti‐ACTB, Alexa Fluor® 488‐conjugated anti‐rabbit IgG, HRP‐linked goat anti‐rabbit IgG, HRP‐linked horse anti‐mouse IgG. Anti‐ATF6, anti‐XBP1, anti‐ChAT, and anti‐AChE antibodies were purchased from Abcam (Cambridge, MA). Anti‐MT1A and anti‐MT1B antibodies were obtained from Santa Cruz Biotechnology (Santa Cruz, CA, USA). Anti‐p‐IRE1α and anti‐GAPDH antibodies were purchased from Novus Biologicals (Littleton, Colorado, USA) and Beijing Biosynthesis Biotechnology Co., LTD. (Beijing, China), respectively.

### 2.2 Animal experiments

Male C57BL/6J mice were purchased from Guangdong Medical Laboratory Animal Center (Guangzhou, China). All mice were housed under standard laboratory conditions (indoor temperature 21 ± 2°C, relative humidity 55 ± 5%, 12 h light/dark cycle) and allowed ad libitum access to water and food. 8‐week‐old mice (weighing 25–30 g) were randomly divided into four groups (*n* = 10 per group): Con (control); SCOP (scopolamine 4 mg/kg); Mel‐L (scopolamine 4 mg/kg + melatonin 10 mg/kg); Mel‐H (scopolamine 4 mg/kg + melatonin 20 mg/kg). Mice were administered intragastrically with melatonin once a day for 4 weeks. Intraperitoneal injection of SCOP was half an hour before the animal behavior test (Figure S1A). In addition, the control group was given normal saline. These treatments were performed at 8:00–11:00 AM of the day. The final behavioral tests were performed with sevens mice in the control group and 10 in the other experimental groups. The day after the behavioral tests were completed, three to five mice in each group were randomly selected, anesthetized with isoflurane, and then severed to remove the brain. Double‐blind was employed in this study. The operators did not know the group information of the mice during the behavioral tests and sample collection. The process of removing the brain was carried out on ice. The movement should be rapid, and the tissues were put into the refrigerator for cryopreservation at −80°C immediately after removal for the kits and Western blot detection. In addition, three mice in each group were perfused with 4% paraformaldehyde after anesthesia. The brain tissues were removed after fixation and then prepared into paraffin sections for room temperature storage after gradient ethanol (30%, 50%, 70%, 80%, 95%, and 100%) dehydration, xylene transparency, and thermostat oven permeabilization, embedding in the embedding machine, sectioning machine (hippocampal coronal section, 4–6 μm), spreading, filleting, patching, and baking, and then stored at room temperature, for Nissl staining and immunofluorescence detection.

### 2.3 Morris water maze test

The SCOP was injected 30 min before the behavioral tasks, while melatonin was administrated as usual. Each mouse was trained from four water entry points 1 day for three consecutive days. The escape latency was recorded for each trail for five consecutive days. On the sixth day, the spatial probe test was executed. Each mouse was allowed to swim for 60 s without the platform. The time spent in the platform quadrant, the number of crossings where the platform was before, and the average speed of mice swimming were measured.

### 2.4 Open field test

The open field test was conducted as reported by Yu Xi et al^47^ with minor modifications. The numbers and distance of each mouse entered the central area (the middle four squares) and the total distance they traveled were recorded, respectively.

### 2.5 Novel object recognition test

In the habituation session, mice were allowed to explore the chamber for 5 min. In the familiarization session, two identical objects were placed in an open arena (30 cm × 30 cm) where each mouse was allowed to move freely for 5 min. In the test session, one of the objects was replaced with a new one (the other one was still day one), and the mice were allowed to explore freely for 5 min again, provided that all mice explored both objects for a total of greater than 20 s. Exploration times of novel and familiar objects were recorded to calculate the discrimination index (the ratio of exploration time of the novel object to total exploration time).

### 2.6 ^18^F‐FDG PET imaging

The assay was performed 4 weeks after drug administration, and the mice were fasted at 23:00 the night before the assay, and the assay was started at 8:00 AM The next day to ensure that the mice were fasted for more than 8 h. The mice were first injected intraperitoneally with 10% chloral hydrate at a volume of 0.1 mL/10 g. After the mice were anesthetized, ^18^F‐FDG developer (120 μCi, radiopure >95%) was injected via the tail vein, and the anesthetized mice were immobilized in the limbs after 45 min. Then, it was scanned by Focus 220 microPET scanner (Siemens Medical Solutions USA, Inc., Knoxville, TN, USA). The dynamic scans were conducted for 0.5 h. PET images were reconstructed by using the microPET‐CT manager (Siemens Medical Solutions USA, Inc.).

### 2.7 Nissl staining

The Nissl staining was conducted according to those we reported earlier.^48^ Brain paraffin sections were successively treated with xylene, alcohol, and distilled water. Then, the sections were stained for 10 min and cleared with distilled water. Images were acquired by microscope (Leica Microsystems, Wetzlar, Germany).

### 2.8 Measurement of the activities of Ach, AChE, ChAT, and Oxidative stress biomarkers

The homogenate of mice brains was centrifuged 12,000*× g* at 4°C for 15 min, and the supernatant was used to detect the levels of acetylcholine (Ach), acetylcholinesterase (AChE), choline acetyltransferase (ChAT), and oxidative stress biomarkers. Their absorbance can be measured by a Universal Microplate Spectrophotometer (Bio‐Rad, Hercules, CA, USA).

### 2.9 Western blotting analysis

RIPA with phosphatase and protease inhibitors was used to lyse mice brains or HT22 cells. The following primary antibodies were used: Metallothionein 1A (MT1A), Metallothionein 1B (MT1B), AChE, ChAT, brain‐derived neurotrophic factor (BDNF), postsynaptic density protein‐95 (PSD95), SIRT1, Bip, protein disulfide isomerase (PDI), PERK, phosphorylate PERK (p‐PERK), IRE1α, phosphorylate IRE1α (p‐IRE1α), activating transcription factor 6 (ATF6), and X‐box binding protein 1 (XBP1).

### 2.10 Immunofluorescence

The tissue sections or cell slides were fixed with 4% paraformaldehyde for 30 min, permeabilized with 0.5% Triton X‐100 for 15 min, and washed with PBS for 3 × 3 min. Then, they were blocked with 10% bovine serum albumin (BSA) and dropped the primary antibodies (SIRT1 1:400, p‐IRE1α 1:400, XBP1 1:400) at 4°C overnight. The Alexa Fluor® 488‐conjugated anti‐rabbit (1:1000) was used as a secondary antibody and sealed with DAPI. The samples were photographed by using a fluorescence microscope (Leica Microsystems, Wetzlar, Germany).

### 2.11 Cell culture and treatment

HT22 cells were incubated in Dulbecco's modified Eagle medium (DMEM) with 10% fetal bovine serum (FBS) and 1% Penicillin–Streptomycin (P/S) at 37°C in air containing 5% CO_2_. The media was changed daily, and the cells were successively used when they reached 80% fusion. The cells were incubated in a serum‐free medium with melatonin for 24 h in a 96‐well plate, then treated with SCOP^49^ or TM^50^ for another 24 h.

### 2.12 RNAi transfection

HT22 cells were transfected with *Sirt1* RNAi (50 nM) or control RNAi (50 nM) by using a Lipofectamine 3000 transfection reagent. Transfection occurred 24 h before the addition of other treatments.

### 2.13 Statistical analysis

SPSS26.0 software was used for statistical analysis, and GraphPad Prism8 software was used for data visualization, and *p* < 0.05 suggested that the difference between groups was statistically significant. Since the sample size of this study was less than 100, the Shapiro–Wilk method was used to test whether the data of each group met the normal distribution. If it met the normal distribution, the data were described by mean ± SD, and the one‐way ANOVA was used to detect the difference between the groups of each index. If there was a difference between the groups, the Bonferroni method was used to compare two by two when the variance was uniform. When the variance was not uniform, the Bonferroni method was used to compare two by two. In this study, all experimental data were statistically tested using the above method except for the escape latency data of the navigation test, which had to be tested using analysis of variance of repeated measures data.

## 3 RESULTS

### 3.1 Melatonin protects against SCOP‐induced learning and memory impairment

**FIGURE 1:**
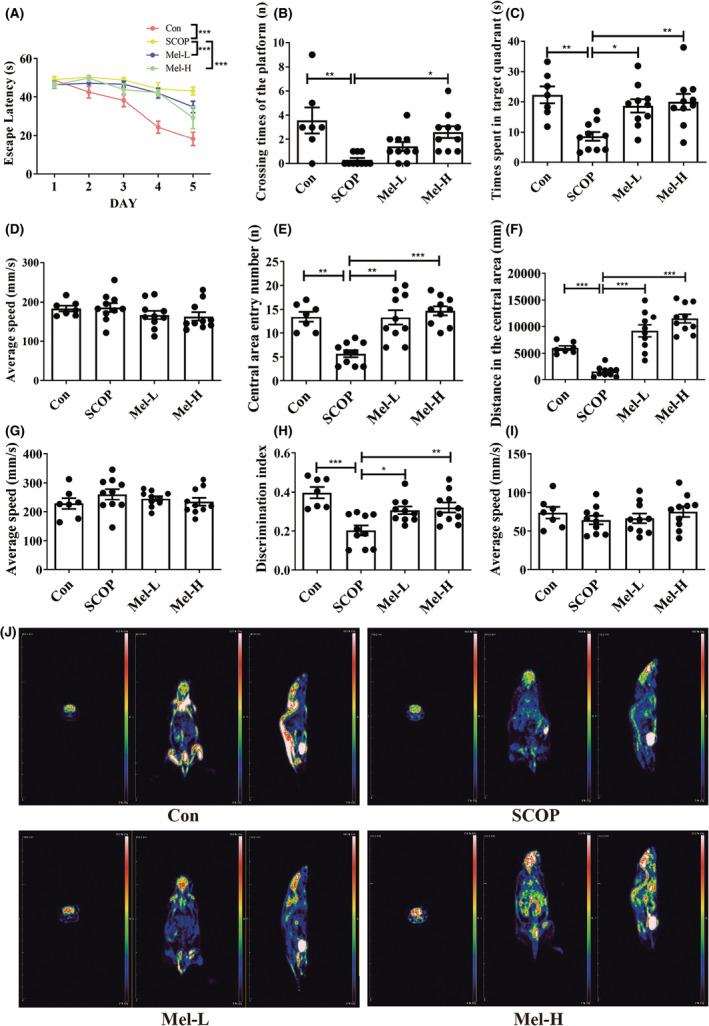
Melatonin protects against SCOP‐induced learning and memory impairment. The escape latency to find the platform (A), crossing times of the platform (B), times spent in the target quadrant (C), and average speed (D). Central area entry numbers (E), distance in the central area (F), average speed (G). Discrimination index (H), average speed (I), ^18^F‐FDG PET images (J). (*n* = 7 control group and *n* = 10 other per group; *p < 0.05, **p < 0.01, and ***p < 0.001). The bars indicate SEM.

To evaluate spatial learning and memory, we performed the Morris water maze test. The average escape latency of each group decreased after training. However, the SCOP group displayed greater escape latency to find the platform, less time crossing the platform, and shorter time spent in the target quadrant than the control group. Melatonin (10 or 20 mg/kg) significantly improved the performances of SCOP‐treated mice in the Morris water maze test (Figure 1A–C; Figure S1B, *n* = 7 control group, *n* = 10 other per group). There was no difference in the average swimming speed among these groups (Figure 1D). To test the condition of anxiety (mice with high anxiety levels spend more time on the periphery and less time in the center),^51^ the open field test was performed. When placed in the open field area, the mice of the SCOP group traveled shorter distances in the central region than those in the control group. However, the mice in the melatonin groups traveled longer distances in the central area compared to those in the SCOP group (Figure 1E,F; Figure S1C). There was no difference in the average speed among the groups (Figure 1G). We also conducted a novel object recognition test to evaluate the recognition memory of mice. Compared to the control group, the mice of the SCOP group exhibited deficits in recognition memory, while the mice in melatonin groups showed an enhanced recognition memory (Figure 1H; Figure S1D). There was no difference in the average speed in each group (Figure 1I). Dementia is characterized by decreased glucose metabolism in the brain. ^18^F‐FDG‐PET imaging was used to evaluate the cerebral glucose uptake of the mice. As shown in Figure 1J, results suggested that ^18^F‐FDG uptake intensity was decreased in the brain of SCOP mice. Melatonin treatment remarkably increased the ^18^F‐FDG uptake of SCOP‐treated mice. Thus, these results indicated that melatonin could protect mice against SCOP‐induced memory deficits.

### 3.2 Melatonin ameliorates oxidative stress, cholinergic nerve system dysfunction, and neurodegeneration in SCOP‐treated mice

**FIGURE 2:**
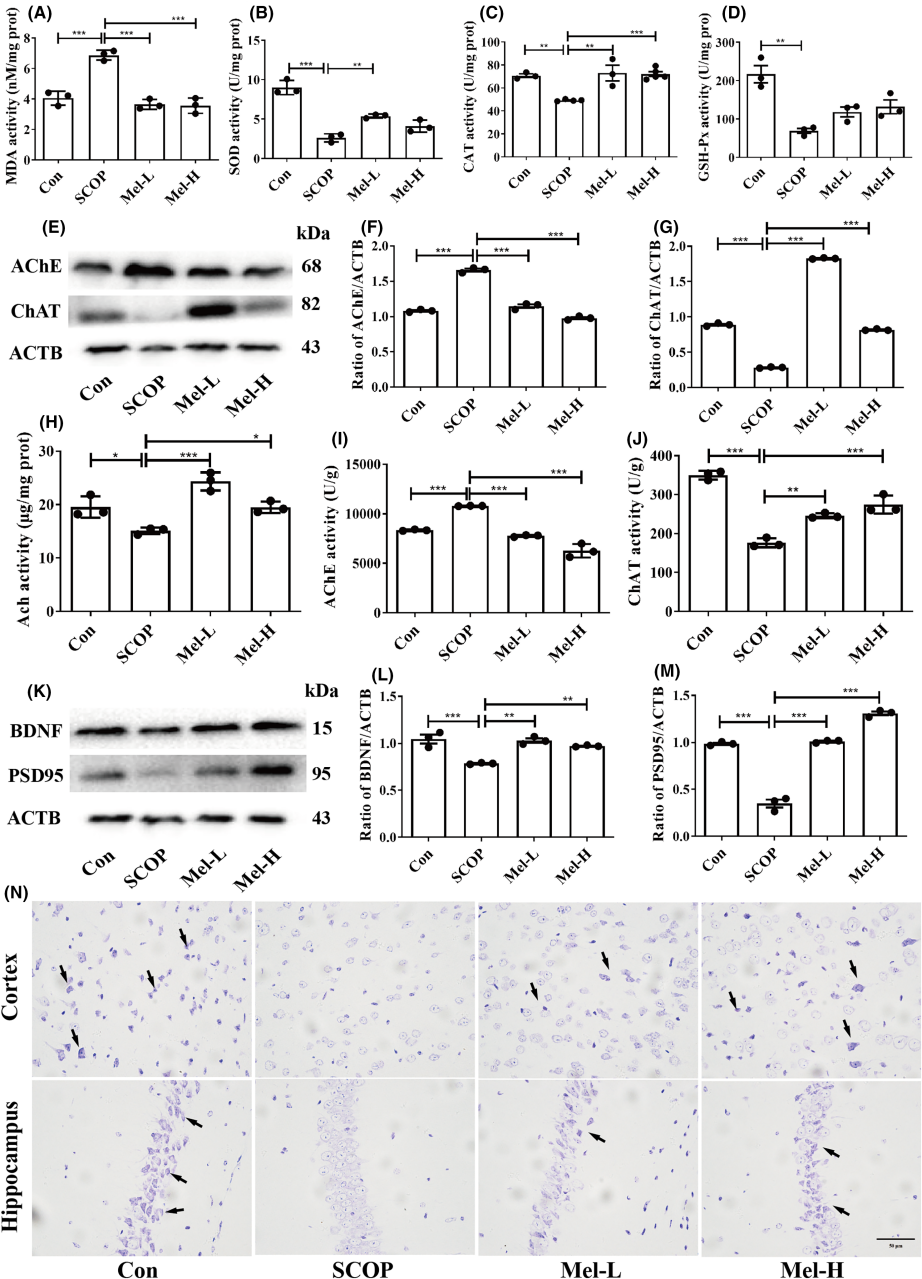
Melatonin ameliorates oxidative stress, cholinergic nerve system dysfunction, and neurodegeneration in SCOP‐treated mice. Effects of melatonin on the activities of MDA (A), SOD (B), CAT (C), and GSH‐Px (D). Representative pictures of the Western blot bands of AChE, ChAT, and ACTB (E–G). Effects of melatonin on the activities of Ach (H), AChE (I), and ChAT (J). (*p < 0.05, **p < 0.01, and ***p < 0.001). The bars indicate SEM. Representative pictures of the Western blot bands of BDNF, PSD95, and ACTB (K‐M). Nissl staining diagram of each group (N). Scar bar = 50 μm.

We first detected the oxidative stress in mice's brains. The results showed that SCOP treatment significantly elevated MDA levels and reduced the activities of antioxidant enzymes (SOD, CAT, and GSH‐Px) when compared to the control group. However, melatonin repressed MDA production and increased SOD and CAT activities (Figure 2A–C). Melatonin treatment increased GSH‐Px activity, although there was no statistical difference (Figure 2D). Then, the levels of Ach, AChE, and ChAT were detected to reveal the effect of melatonin on the cholinergic nerve system. In the SCOP group, the AChE level was increased, while Ach and ChAT were significantly decreased compared to those in the control group. After melatonin treatment, the AChE level was markedly reduced, and the levels of Ach and ChAT were increased (Figure 2E–J). To study the effect of melatonin on neurotrophic activity and neurodegeneration, neurotrophic factors and Nissl's staining were conducted. The expressions of BDNF and PSD95 were significantly decreased in the SCOP group, but dealing with melatonin increased the levels of BDNF and PSD95 (Figure 2K–M). Nissl's staining further showed that the neurons were weakly stained, and the Nissl bodies were lost in the SCOP group. However, after melatonin treatment, the neurons were regularly arranged and deeply stained (Figure 2N). These results indicated that melatonin could prevent oxidative stress, cholinergic nerve system dysfunction, and neurodegeneration in SCOP‐treated mice.

### 3.3 Melatonin upregulates SIRT1 expression in the hippocampus of SCOP‐treated mice and SCOP‐treated HT22 cells

**FIGURE 3:**
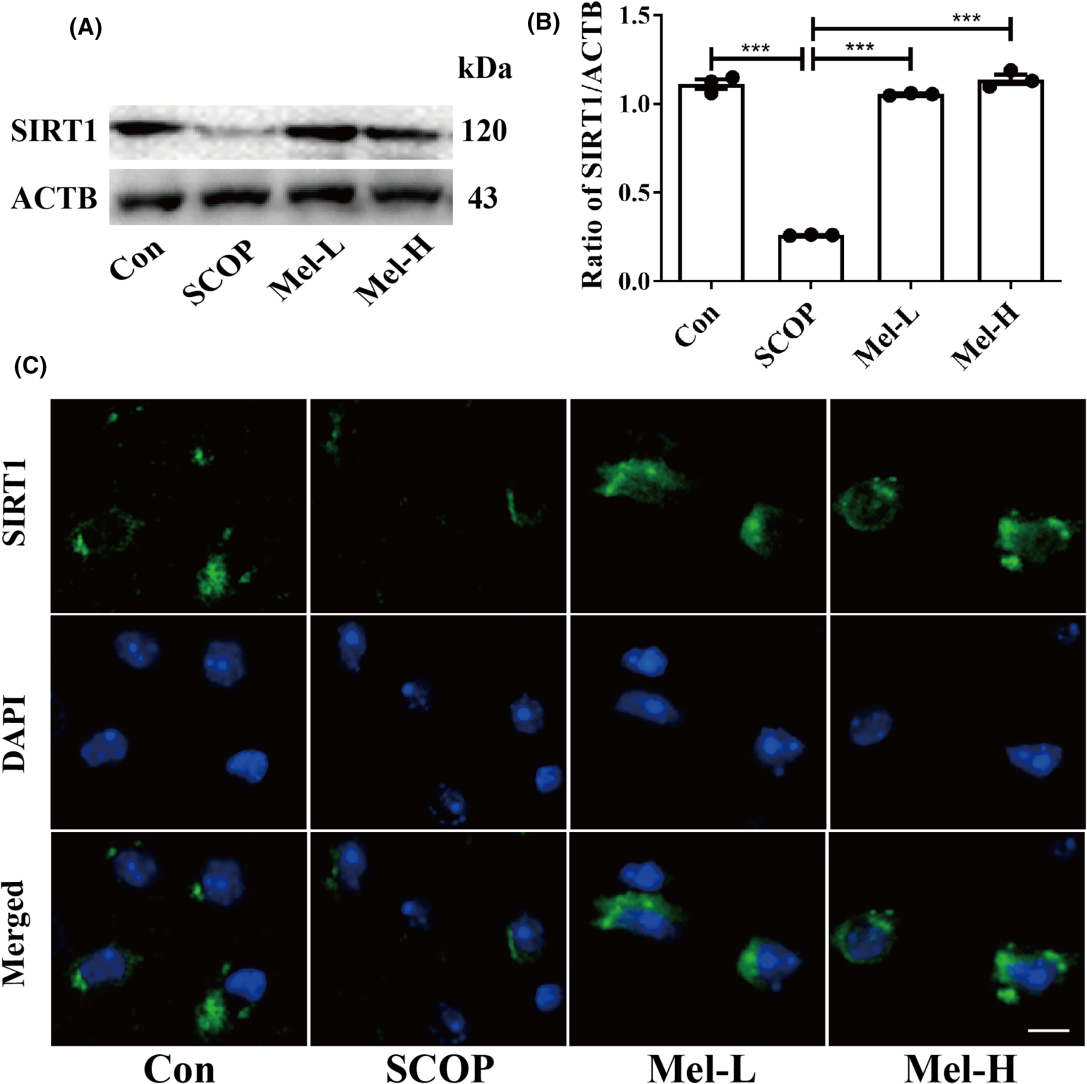
Melatonin upregulates SIRT1 expression in the hippocampus of SCOP‐treated mice. (A, B) Effects of melatonin on the SIRT1 protein by Western blot (*p < 0.05, **p < 0.01, and ***p < 0.001). The bars indicate SEM. The expression of SIRT1 (C) by immunofluorescence. Scar bar = 50 μm.

Firstly, we employed a SCOP‐treated HT22 (mouse hippocampal neuron cell line) cell model to screen the effect of melatonin on the mRNA expressions of the SIRTs family. We selected the dosage of 200 μM melatonin for further study (Figure S2A–D). Then, we found that melatonin effectively increased the expressions of MT1A and MT1B (melatonin receptors) in HT22 cells (Figure S3A–C). In addition, the mRNA expressions of the SIRTs family (Sirt1‐7) were screened, and *Sirt1* expression was obviously increased after melatonin treatment in HT22 cells (Figure S3D). Western blot results also indicated that melatonin increased SIRT1 expression significantly in the hippocampus of SCOP‐treated mice (Figure 3A,B). Immunofluorescence results further showed the same trend (Figure 3C). These results suggested that melatonin could upregulate SIRT1 expression in the hippocampus of SCOP‐treated mice.

### 3.4 Melatonin inhibits p‐IRE1α/XBP1 pathway by activating SIRT1 in the hippocampus of SCOP‐treated mice

**FIGURE 4:**
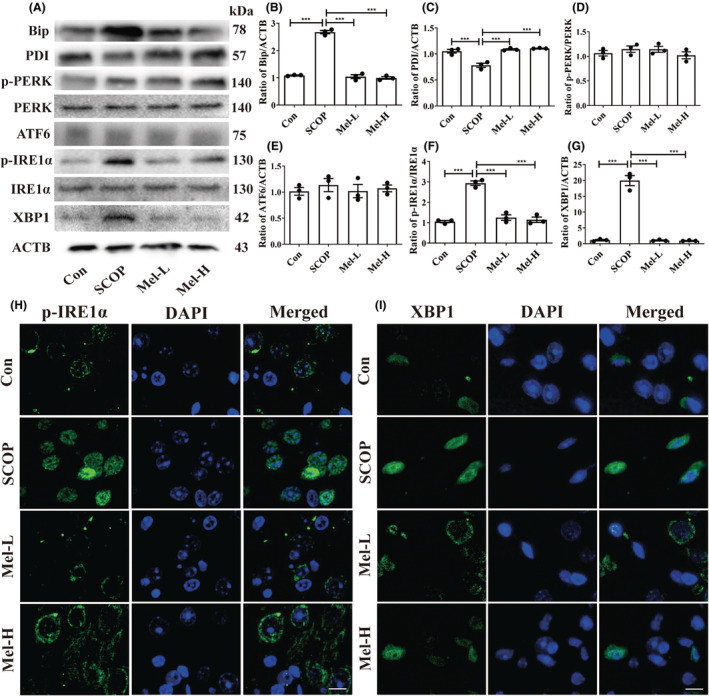
Melatonin inhibits IRE1α/XBP1 pathway in the hippocampus of SCOP‐treated mice. (A‐G) Effects of melatonin on the Bip (B), PDI (C), p‐PERK (D), ATF6 (E), p‐IRE1α (F), and XBP1 (G) proteins were analyzed by Western blot (*p < 0.05, **p < 0.01, and ***p < 0.001). The bars indicate SEM. The expressions of p‐IRE1α (H) and XBP1 (I) in each group via immunofluorescence. Scar bar = 50 μm.

**FIGURE 5:**
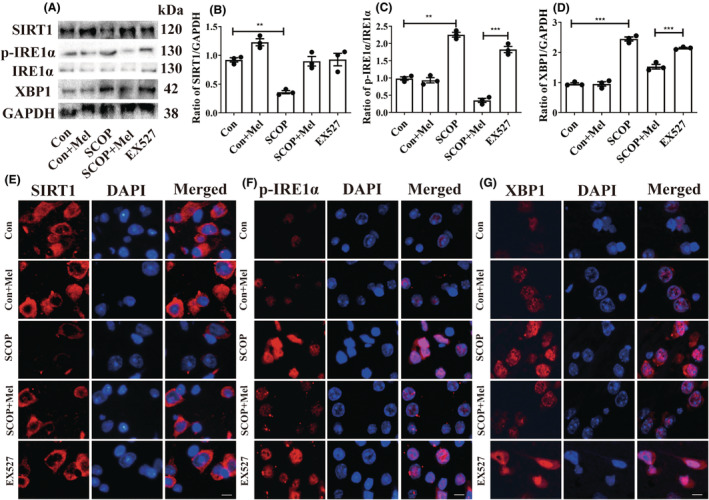
Melatonin inhibits the IRE1α/XBP1 pathway by activating SIRT1 in the hippocampus of SCOP‐treated mice. (A–D) Effects of melatonin on the SIRT1 (B), p‐IRE1α (C), and XBP1 (D) proteins were analyzed by Western blot (*p < 0.05, **p < 0.01, and ***p < 0.001). The bars indicate SEM. The expression of SIRT1 (E), p‐IRE1α (F), and XBP1 (G) by immunofluorescence. Scar bar = 50 μm.

We next measured the protein expressions of ER stress markers, Bip, and PDI, and three ER stress sensors (PERK, IRE1α, and ATF6). The increased Bip level and decreased PDI level were observed in the hippocampus of SCOP‐treated mice. Melatonin administration decreased the Bip level and increased the PDI level (Figure 4A–C). Next, the results showed that only the expressions of phosphorylated IRE1α (p‐IRE1α) and its downstream XBP1 were elevated after SCOP treatment among the three ER stress sensors (Figure 4A,D–G). To further confirm the results, the immunofluorescence method was used to detect the levels of p‐IRE1α and XBP1. Expectedly, the cytoplasmic staining of p‐IRE1α and XBP1 was more intense in the SCOP‐treated group than in the control group (Figure 4H,I). Melatonin treatment effectively decreased the expressions of p‐IRE1α and XBP1 (Figure 4 A,F–I). Furthermore, EX527, which exerts an inhibitory effect on SIRT1 activity but has no impact on SIRT1 expression,^52^ was given to the mice. The EX527 group in Figure 5 was added to the SCOP+Mel group to observe the expression of p‐IRE1α and XBP1 after the inhibition of SIRT1. Both Western blot and immunofluorescence results indicated that when SIRT1 was inhibited by EX527, melatonin could not downregulate the p‐IRE1α/XBP1 axis in SCOP‐induced mice (Figure 5A–G). Our data uncovered a novel mechanism that melatonin attenuates ER stress via the SIRT1/IRE1α/XBP1 pathway in SCOP‐induced mice. Based on these results, we preliminary proved that melatonin decreased the p‐IRE1α/XBP1 axis by upregulating SIRT1 in SCOP‐induced mice.

### 3.5 Melatonin protects against SCOP‐induced injury via SIRT1/IRE1α/XBP1 pathway in HT22 cells

**FIGURE 6:**
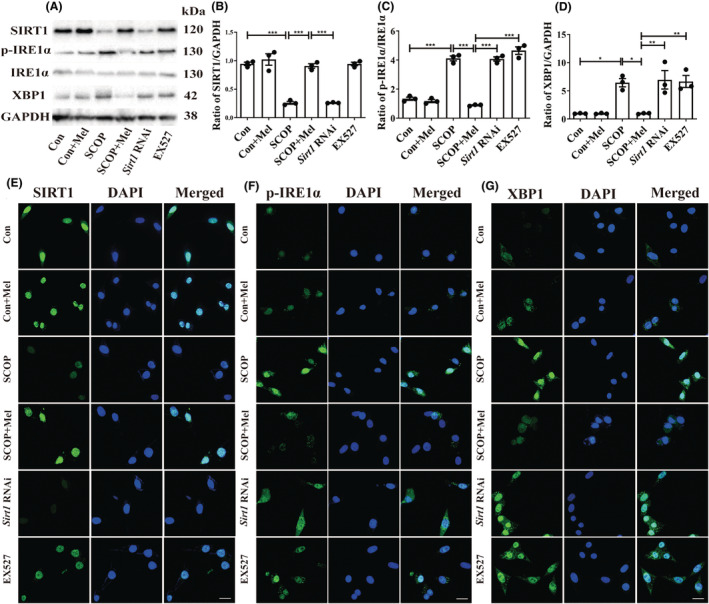
Melatonin protects against SCOP‐induced injury via SIRT1/IRE1α/XBP1 pathway in HT22 cells. (A‐D) Effects of melatonin on the SIRT1 (B), p‐IRE1α (C), and XBP1 (D) proteins were analyzed by Western blot (*p < 0.05, **p < 0.01, and ***p < 0.001). The bars indicate SEM. The expression of SIRT1 (E), p‐IRE1α (F), and XBP1 (G) by immunofluorescence. Scar bar = 50 μm.

Then, we employed SCOP‐treated HT22 cells to verify the pathway. The MTT results indicated that SCOP (4 mM) decreased cell viability, whereas 100 or 200 μM of melatonin could protect against cell damage (Figure S2A–C). Microscopy observation showed that SCOP treatment induced cell shrinkage and irregularly shaped compared with the control group. However, melatonin prevented SCOP‐induced morphological changes (Figure S2D). Next, EX527 and *Sirt1* RNAi (Figure S3E) were used further to verify the relationship between SIRT1 and p‐IRE1α/XBP1 separately. Both siRNA and EX527 are inhibitors of SIRT1, and siRNA and EX527 groups in Figure 6 were added to the SCOP+Mel group to observe the expression of p‐IRE1α and XBP1 after the inhibition of SIRT1. Both Western blot and immunofluorescence results indicated that when SIRT1 was interfered with or inhibited, melatonin could not decrease the expressions of the p‐IRE1α/XBP1 axis in SCOP‐induced HT22 cells (Figure 6A–G). Our data might uncover a novel mechanism that melatonin attenuates ER stress via the SIRT1/IRE1α/XBP1 pathway in SCOP‐induced HT22 cells.

### 3.6 SIRT1 is the upstream of the p‐IRE1α/XBP1 axis in the protective mechanism of melatonin

**FIGURE 7:**
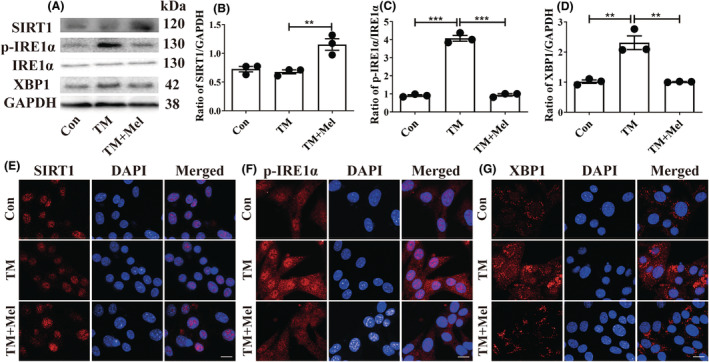
SIRT1 is upstream of the IRE1α/XBP1 axis in the protective mechanism of melatonin. (A–D) Effects of melatonin on the SIRT1 (B), p‐IRE1α (C), and XBP1 (D) proteins were analyzed by Western blot in all groups (*p < 0.05, **p < 0.01, and ***p < 0.001). The bars indicate SEM. The expressions of SIRT1 (E), p‐IRE1α (F), and XBP1 (G) by immunofluorescence. Scar bar = 50 μm.

To further study the upstream and downstream relationship between SIRT1 and p‐IRE1α/XBP1 axis, the TM (ER stress inducer) was used to treat HT22 cells. We chose 0.1 μM TM and 100 μM melatonin as the optional dosages for HT22 cells (Figure S4A–C). The Western blot results showed that TM effectively increased the p‐IRE1α/XBP1 axis expressions while not affecting SIRT1 (Figure 7A–D). Melatonin treatment upregulated SIRT1 expression while decreasing the p‐IRE1α/XBP1 axis in TM‐treated HT22 cells (Figure 7A–D). Immunofluorescence results showed the same trend as Western blot results (Figure 7E–G). Therefore, we concluded that melatonin suppressed p‐IRE1α/XBP1 through activating SIRT1.

## 4 DISCUSSION

In this study, we employed the SCOP‐treated C57BL/6J mice to study the neuroprotective effect of melatonin. The C57BL/6J mice were used to evaluate the effect of exogenous melatonin as a result of endogenous melatonin deficiency due to a point mutation in the AANAT gene.^53^, ^54^ Previous studies have reported that a high melatonin dosage can act as an antioxidant^55^ and inhibit tumor cells,^56^ while also activating serotonin receptors to produce side effects.^57^ We adopted the dosages of 10 and 20 mg/kg melatonin, which were above the physiological level but had been used in mice studies without affecting circadian rhythms.^58^, ^59^ In addition, using high concentrations (micromolar and millimolar) of melatonin on HT22 cells has also been reported,^60^ which is generally associated with its anti‐oxidative effects.^61^ The retrieval of specified memories with stable recollective experiences and recollection‐like memory is benefited by hippocampal function.^62^, ^63^, ^64^ A previous study reported that melatonin could reduce inflammation and neuronal apoptosis in the hippocampus and memory function in mice with sleep deprivation (SD).^65^ Melatonin has also been reported to it could lessen anxiety and depression‐like behaviors of AD mice by regulating the expression of glutathione S‐transferase P1 (GSTP1) and complexin‐1 (CPLX1) in the hippocampus.^66^ In our study, melatonin improved learning and memory to alleviate anxiety‐like behaviors in SCOP‐treated mice and ameliorated oxidative stress damage, cholinergic dysfunction, and neurodegenerative changes. We identified a novel mechanism that melatonin relieved SCOP‐induced cognitive dysfunction through the SIRT1/IRE1α/XBP1 axis. It further supports the view that melatonin acts as a promising therapeutic for preventing cognitive disorders.

SCOP‐induced cognitive defects are primarily related to cholinergic dysfunction.^67^, ^68^, ^69^ Additionally, accumulating evidence indicates that cholinergic dysfunction may underlie autism‐related behavior and anxiety‐related behavior.^70^, ^71^ Some researchers reported melatonin might facilitate the improvement of cognitive function of Down syndrome (DS) animal model and its control (euploid litter mates) mice via descending the age‐associated degeneration of cholinergic neurons partially in basal forebrain.^72^ Melatonin treatment improves cognitive impairments by restoring the cholinergic system in the prefrontal cortex,^73^ which is involved in mood and emotion processing. In this study, melatonin effectively improved cholinergic dysfunction and attenuated anxiety‐like behaviors in mice in the open field test. Neurotrophins, like BDNF and PSD95, have been listed as the primary target affected by SCOP. SCOP‐induced memory impairment can be attenuated by regulating the ERK/CREB/BDNF signaling pathway.^74^ Similarly, the level of BDNF was decreased in SCOP‐induced male rats.^75^ In addition, SCOP can also lead to oxidative stress.^76^, ^77^ In the current study, we detected neurotrophic factors and oxidative stress biomarkers and found that melatonin could enhance the expressions of neurotrophins and suppress oxidative stress in SCOP‐treated mice.

A deficiency of SIRT1 may contribute to nerve cell dysfunction and early memory deficits.^78^ An early report indicated that SIRT1 can suppress intracellular Aβ accumulation in N2aSwe cells.^79^ Pretreatment with melatonin could prevent cognitive defects and neuronal disturbances by strengthening the deacetylation of SIRT1 substrates.^80^ Melatonin had a neuroprotective effect on the dentate gyrus of aged rats through the SIRT1 pathway.^81^ As expected, our results suggested that melatonin could increase the expression of SIRT1 in SCOP‐treated mice. Besides, a recent study reported that MEL crosses the blood–brain barrier and metabolized to one of the MEL's metabolites, N1‐acetyl‐5‐methoxykynuramine (AMK) in the brain, which could ameliorate long‐term memory and be regarded as a potential target point.^82^ However, we did not explore the effect of SIRT1 on melatonin's metabolites in this study. We will consider figuring it out in the future. Moreover, aging could result in less melatonin, which strongly correlates with aging‐associated cognitive dysfunction.^83^ Different administrated points and doses of melatonin, whether contributing to the process of cognition impairment induced by various diseases, would be interesting research directions.

ER stress sensors, like p‐PERK, p‐IRE1α, and ATF6, participate in diabetes mellitus‐induced cognitive dysfunction.^84^ During the development of cognitive impairment, the activation of ER stress is closely associated with synaptic dysfunction, neuronal cell death, and axonal degeneration.^85^ Studies have shown that melatonin is closely related to maintaining ER homeostasis.^86^ Melatonin maintains ER homeostasis in diabetic fatty rats via the IRE1α pathway.^87^ In addition, melatonin attenuates BLM‐induced pulmonary fibrosis by decreasing the expression of p‐IRE1α and its downstream target XBP1 to activate JNK.^88^ In this study, we tested the primary markers of ER stress, Bip, and PDI,^89^ as well as the three ER stress sensors. We found that melatonin mainly inhibited the IRE1α/XBP1 axis. SIRT1 is closely linked with ER stress. SIRT1‐mediated deacetylation acts on alleviating hepatic ER stress in lipid‐induced mice.^90^ SIRT1 protects cardiomyocytes against ER stress‐induced apoptosis via the PERK/eIF2α axis.^91^ A recent study showed that SIRT1 could be essential in reducing hypoxia‐induced apoptosis through the IRE1α pathway.^92^ In addition, SIRT1 deacetylated XBP1s and inhibited its transcriptional activity.^93^

Ex527^94^, ^95^, ^96^ acts as a potent SIRT1 inhibitor, and *Sirt1* RNAi^97^, ^98^ are used in vivo and in vitro studies, including tumorigenesis glucose metabolism, islet function, and so on. In our study, the relationship between SIRT1 and IRE1α/XBP1 axis was certificated by EX527 or *Sirt1* RNAi. Results indicated that melatonin inhibited SCOP‐induced ER stress by regulating the SIRT1/IRE1α/XBP1 pathway. TM‐activated IRE1α/XBP1 axis further verified the phenomenon.

To sum up, our findings, combined with in vivo and in vitro studies, validate that melatonin reduces p‐IRE1α/XBP1 expression by activating SIRT1 in SCOP‐induced cognitive dysfunction. It provides a novel foundation for the development of therapies targeting transient cognitive impairment. It will be interesting to examine whether the melatonin‐mediated SIRT1/IRE1α/XBP1 axis is also beneficial in other cognitive‐related diseases. Furthermore, this establishes a basis for future research on melatonin and the cholinergic system's interaction in cognitive or emotional disorders. There are some limitations in this study. SIRT1 KO mice or IRE1α inhibitors in the future should be used to confirm its mechanism in detail further.

## AUTHOR CONTRIBUTIONS

S.Z. conceived and designed the study. X.L. and S.H. performed the experiments. C.W. and T.H. helped with the experiments. Y.C. and Q.W. supported the materials. X.L., J.Z., and S.Z. wrote and revised the manuscript. S.Z. acquired funding support and approved the final manuscript as submitted.

## FUNDING INFORMATION

This work was supported by the National Natural Science Foundation of China (No. 82004430, 82174310), Guangdong Basic and Applied Basic Research Foundation (No. 2023B1515120075), and Guangdong Provincial Key Laboratory of Research on Emergency in TCM (No. 2023B1212060062).

## CONFLICT OF INTEREST STATEMENT

The authors declare no competing interests.

## CONSENT

Not applicable.

## Supporting information


Data S1.

Data S2.

## Data Availability

The data that support the findings of this study are available from the corresponding author upon reasonable request.
