# Supplementary material for: Melatonin attenuates scopolamine‐induced cognitive dysfunction through SIRT1/IRE1α/XBP1 pathway
Source: CNS Neurosci Ther. 2024 Jul 26;30(7):e14891. doi: 10.1111/cns.14891 (PMC11273216; doi:10.1111/cns.14891)
Supplement: Supplementary file 1 — Data S1. [file CNS-30-e14891-s001.doc]

**Supplementary Materials and Methods**

**Measurement of cell viability**

The 10 % MTT solution replaced the medium with different treatments for 4 h. Then, removed the MTT solution, added the DMSO. After the formazan crystals were completely dissolved, the absorbance was measured at 570 nm by Universal Microplate Spectrophotometer (Bio-Rad, Hercules, CA, USA). The rate of cell viability was calculated (OD value of treatment groups / OD value of control group × 100 %).

**Real-time quantitative PCR**

Total RNA was isolated from HT22 cells using RNAiso Plus, chloroform, isopropanol in turn and the concentration of RNA was measured by NanoDrop 2000/2000c (Thermo Fisher Scientific, Rockford, IL, USA). The mRNA was converted into cDNA using Prime Script RT Master Mix through a reverse transcription reaction process. PCR reaction was executed with Applied Biosystems 7500 Real-Time PCR System (Thermo Fisher Scientific, Rockford, IL, USA) by using TB Green Premix Ex Taq. The primer sequences were as follows: *Sirt1* forward, 5’-TGGTTCCAGTACTGCAGACA-3’, reverse, 5’-GTATACCTCAGCACCGTGGA-3’; *Sirt2* forward, 5’-GAGCCGGACCGATTCAGAC-3’, reverse, 5’-GGAGCGGAAGTCAGGGATAC-3’; *Sirt3* forward, 5’-GATTCGGATGGCGCTTGAC-3’, reverse, 5’-TCTCCCACCTGTAACACTCCC-3’; *Sirt4* forward, 5’-AGACCCATCCAGCACATTGA-3’, reverse, 5’-TCCACGTTCTGAGTCACCAA-3’; *Sirt5* forward, 5’-CATTCTGGAGGAGGTGGACA-3’, reverse, 5’-GGGTCCGGGAAAATGAAACC-3’; *Sirt6* forward, 5’-GGGGCTGTCAGTCTTTGTTG-3’, reverse, 5’-GCTCCGTTAACATGAGGCAG-3’; *Sirt7* forward, 5’-AGAAATATCCCCGCCTCTGG-3’, reverse, 5’-GAAAGTCTTCAGGGCAGCAG-3’; *ACTB* forward, 5’-GATCAAGATCATTGCTCCTCCTG-3’, reverse, 5’-AGGGTGTAAAACGCAGCTCA-3’.

**Supplementary Fig. 1.**

**
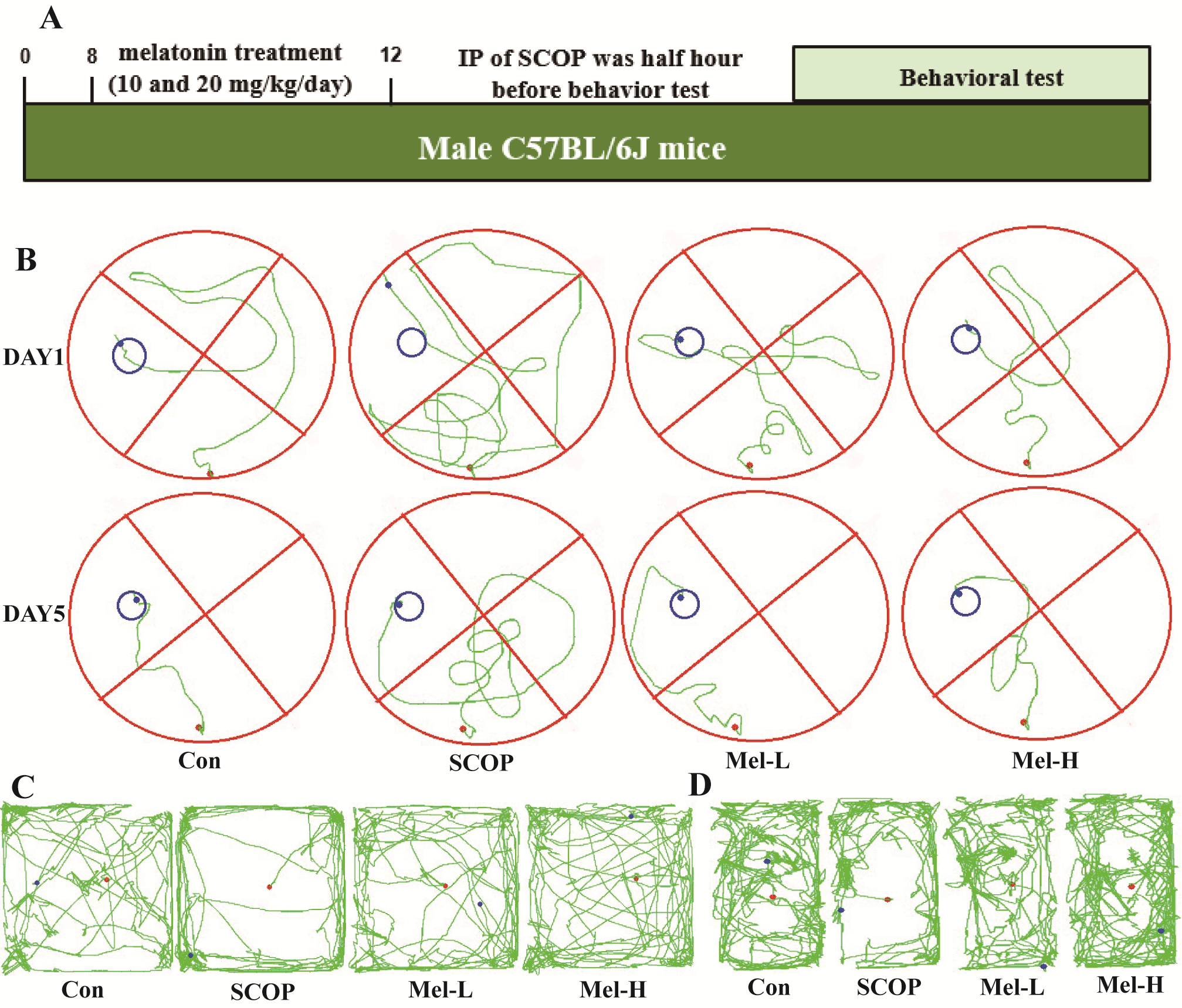
**

**Supplementary Fig. 1.** The trajectory of animal behavior. The timeline of animal experiments (A), track diagram of DAY1 and DAY5 (B), trajectory map of exploration (C) and trajectory map of learning (D).

**Supplementary Fig. 2.**


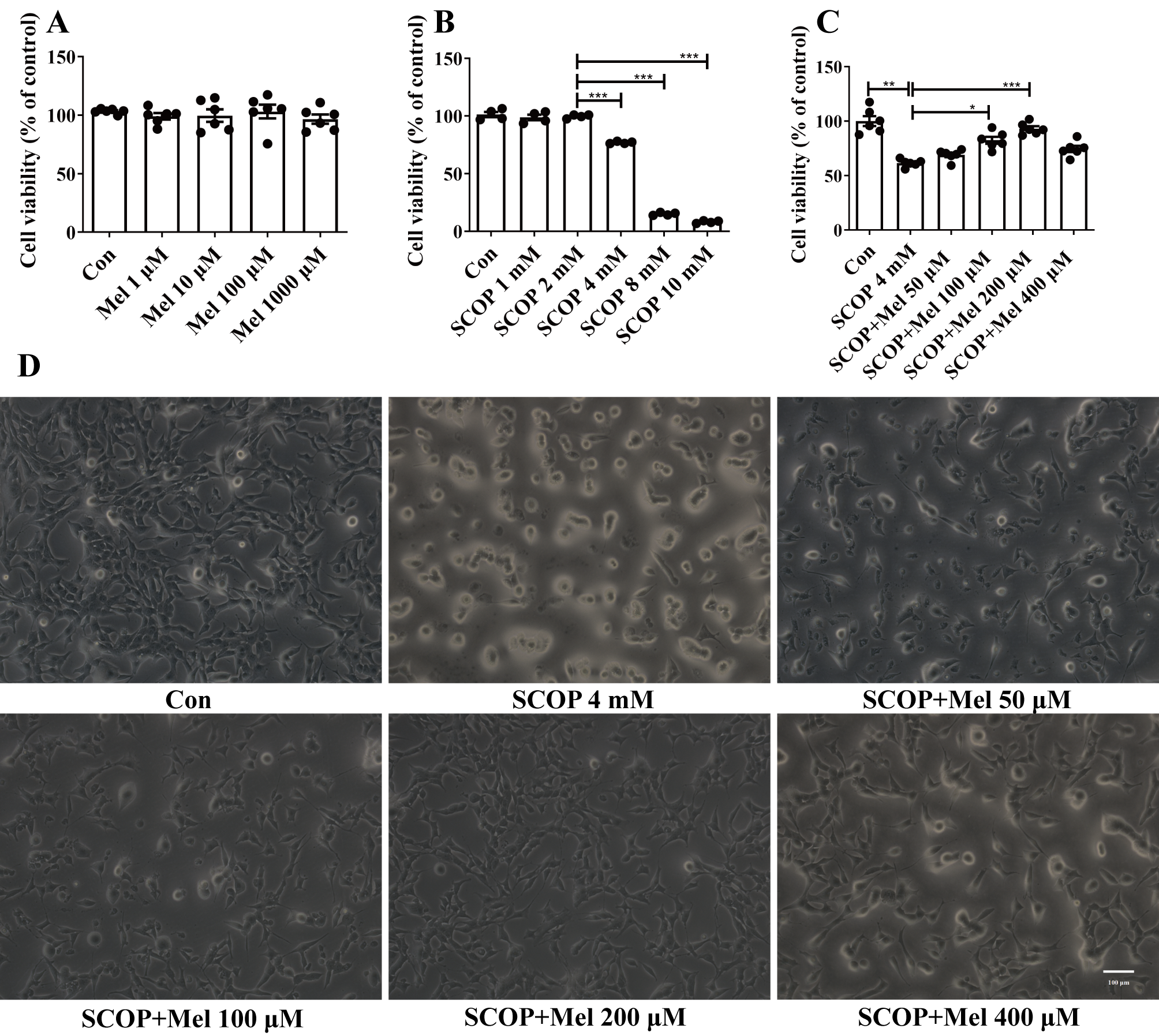


**Supplementary Fig. 2.** Effects of melatonin on SCOP-induced HT22 cell death. The cell viability after different concentrations of melatonin (24 h) (A), SCOP (24 h) (B) and adding melatonin for 24 h and then with or without SCOP (4 mM) for 24 h (C) (**p* < 0.05, ***p* < 0.01, and ****p* < 0.001). The bars indicate SEM. Morphological changes of SCOP-induced HT22 cells with or without melatonin (D). Scar bar = 100 μm.

**Supplementary Fig. 3.**


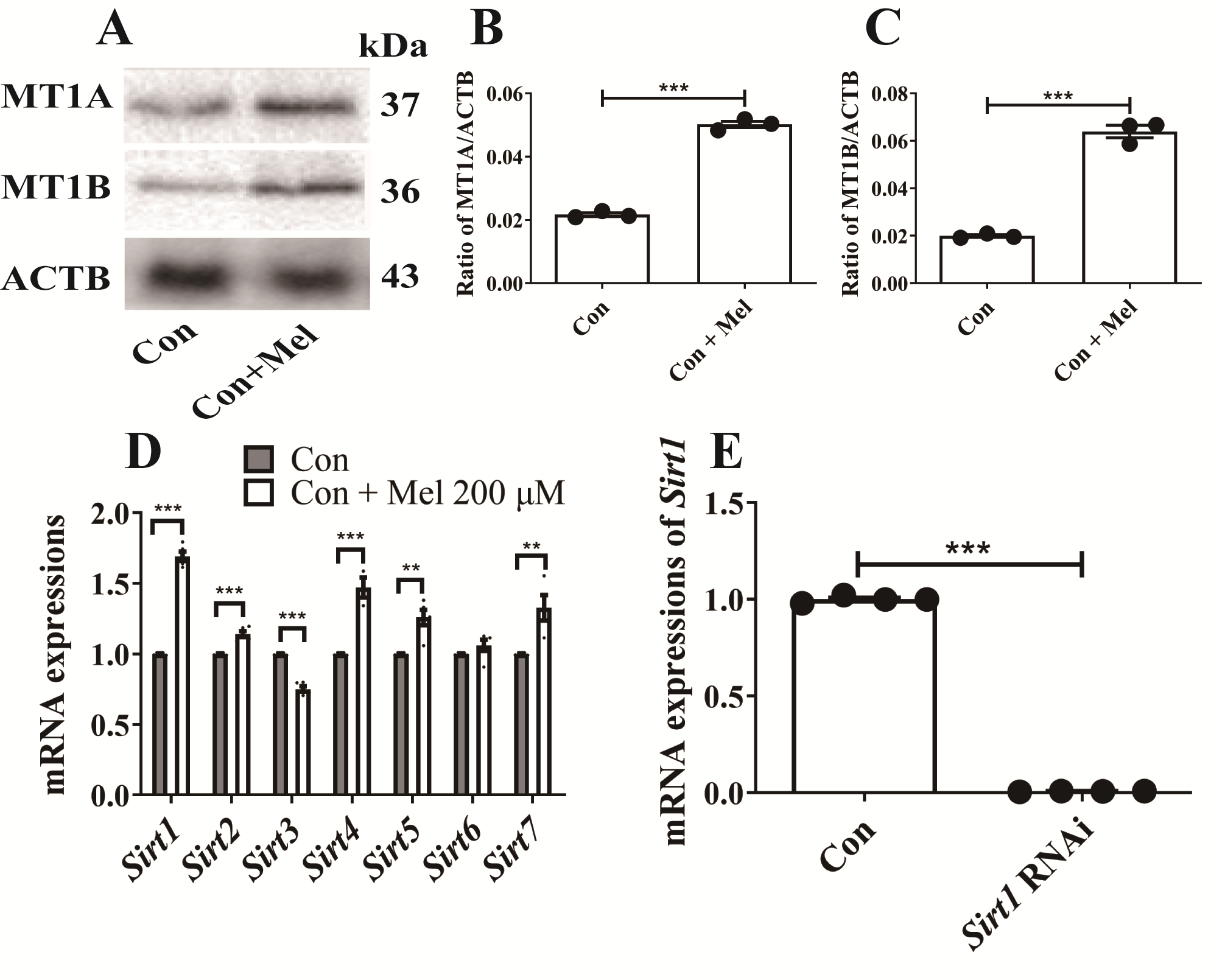


**Supplementary Fig. 3.** The expression of melatonin receptors and sirtuins family in melatonin-treated HT22 cells. (A-C) Expression of MT1A (B) and MT1B (C) proteins in melatonin-treated HT22 cells by western blot. The relative expression of *Sirt1-7* mRNAs was analyzed by quantitative RT-PCR (D). The mRNA expression of *Sirt1* after adding *Sirt1* RNAi 24 h by quantitative RT-PCR (E) (**p* < 0.05, ***p* < 0.01, and ****p* < 0.001, *vs.* control). The bars indicate SEM.

**Supplementary Fig. 4.**


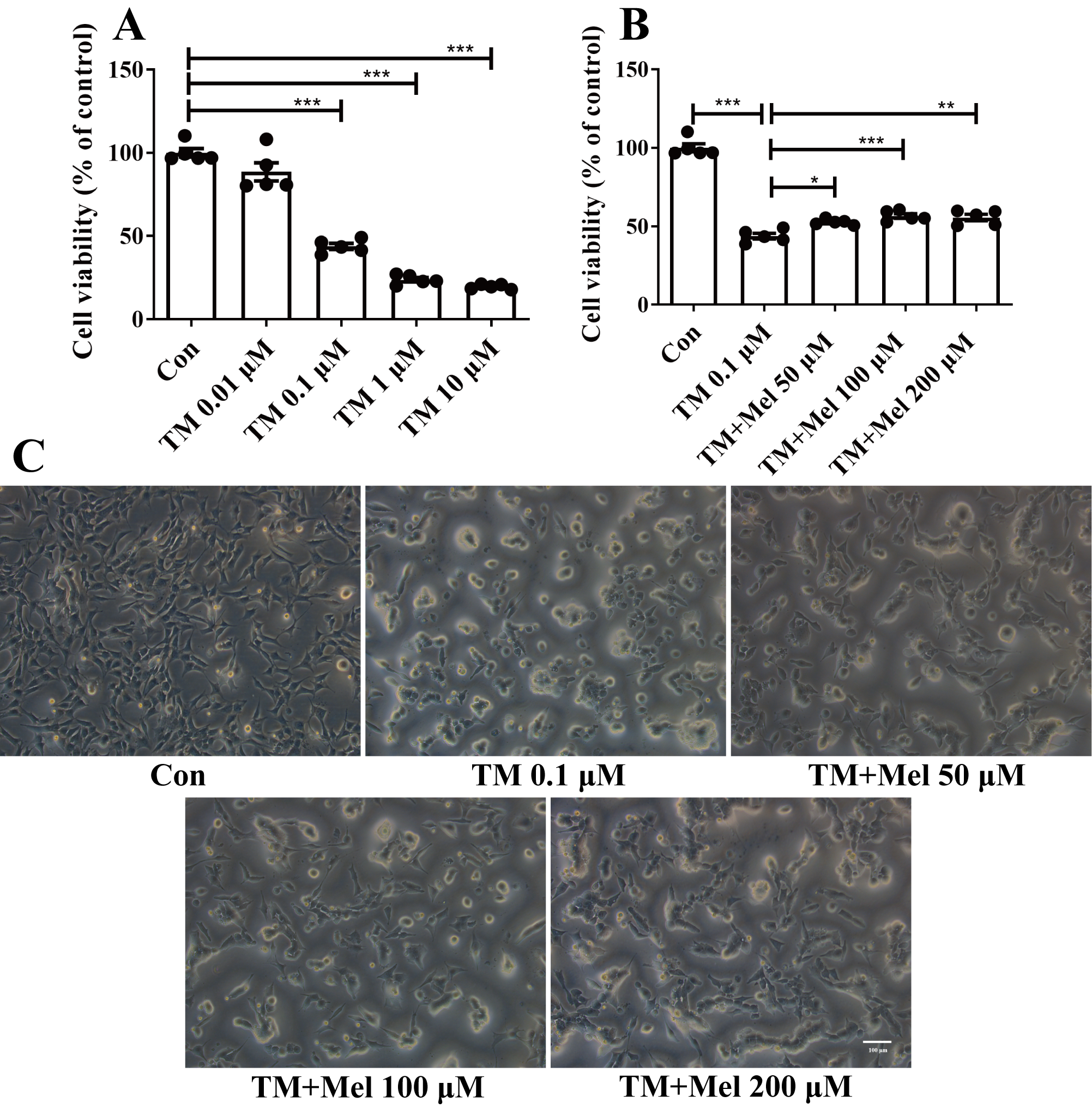


**Supplementary Fig. 4.** Effects of melatonin on TM-induced HT22 cell death. The cell viability after different concentrations of TM (24 h) (A) and adding melatonin for 24 h and then with or without TM (0.1 μM) for 24 h (B) (**p* < 0.05, ***p* < 0.01, and ****p* < 0.001). The bars indicate SEM. Morphological changes of SCOP-induced HT22 cells with or without melatonin (C). Scar bar = 100 μm.
