# Supplementary material for: Melatonin attenuates scopolamine‐induced cognitive dysfunction through SIRT1/IRE1α/XBP1 pathway
Source: CNS Neurosci Ther. 2024 Jul 26;30(7):e14891. doi: 10.1111/cns.14891 (PMC11273216; doi:10.1111/cns.14891)
Supplement: Supplementary file 2 — Data S2. [file CNS-30-e14891-s002.pdf]

Figure 2

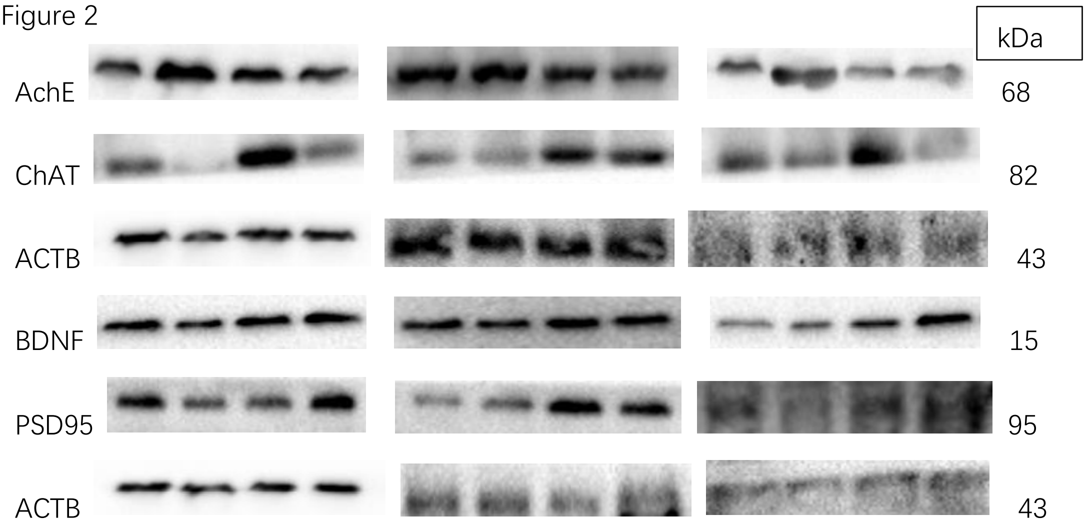


The original western blots of Figure 2.

Figure 3

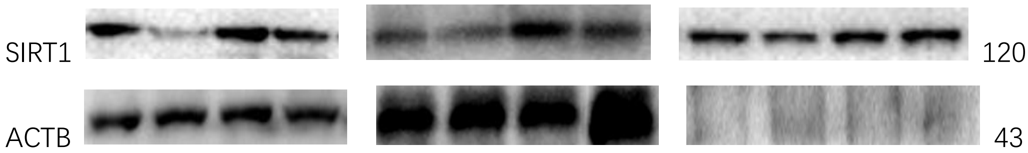


The original western blots of Figure 3.

Figure 4

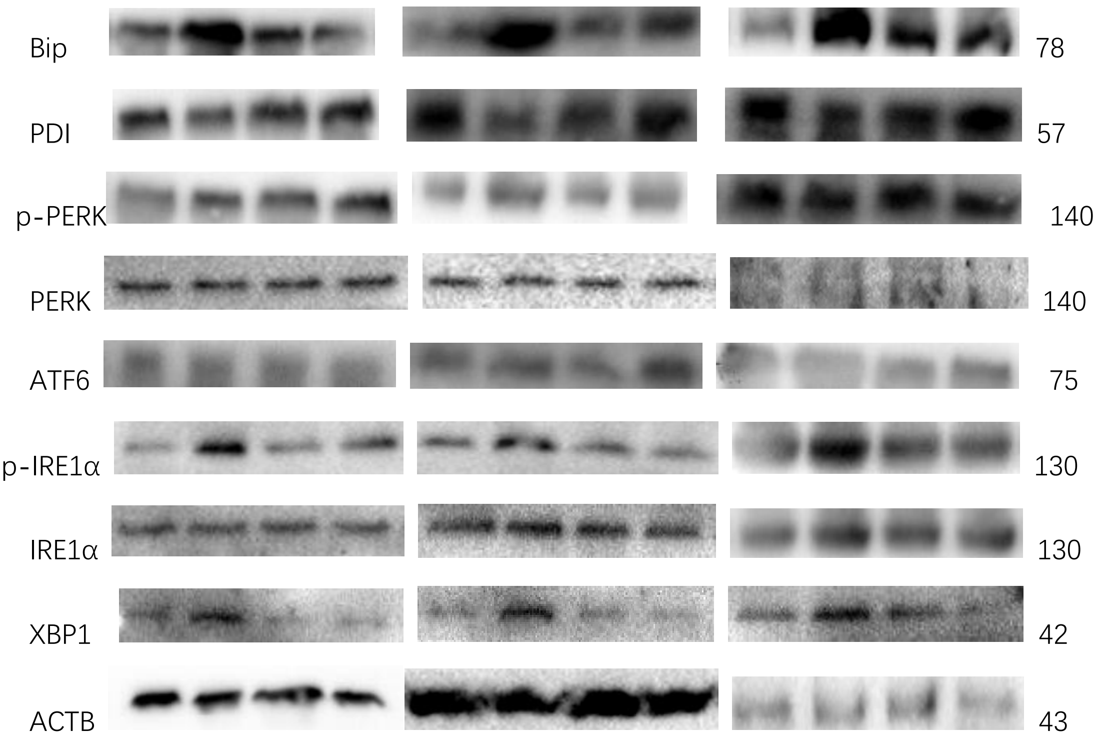


The original western blots of Figure 4.

Figure 5

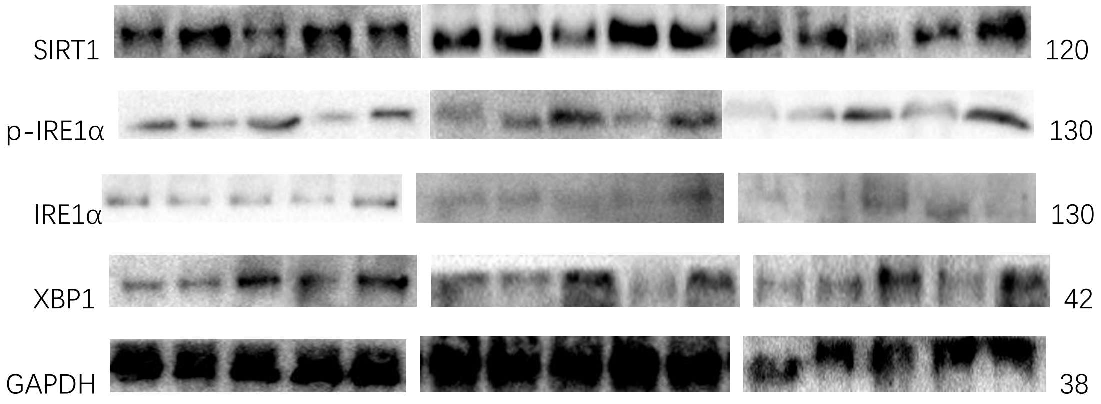


The original western blots of Figure 5.

Figure 6

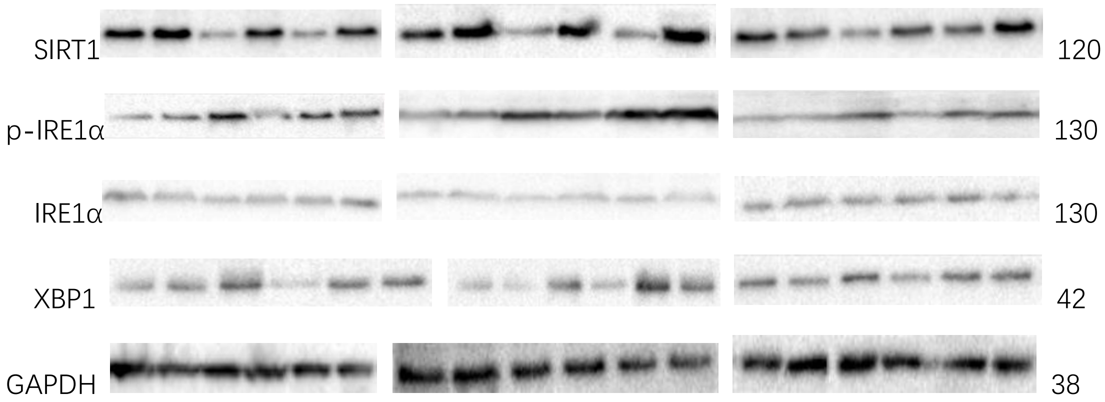


The original western blots of Figure 6.

Figure 7

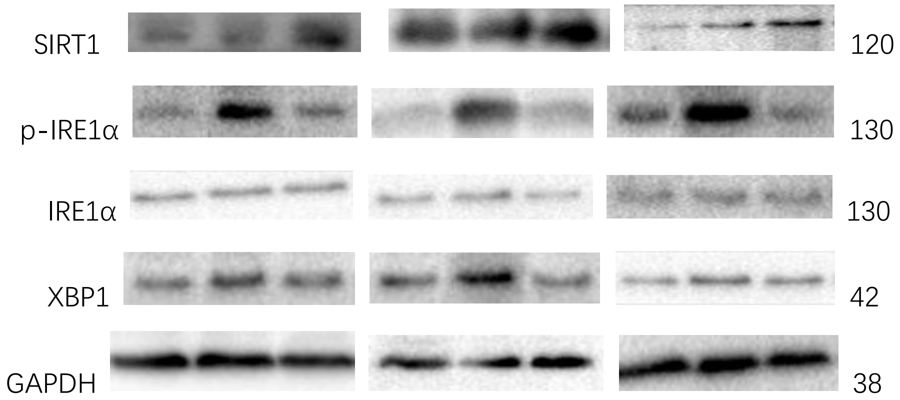


The original western blots of Figure 7.

Supplementary Figure 3

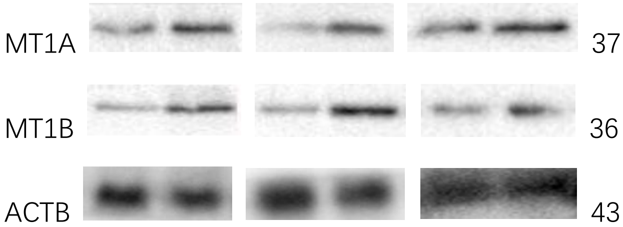


The original western blots of Supplementary Figure 3.
